# Research in epidemic and emergency situations: A model for collaboration and expediting ethics review in two Caribbean countries

**DOI:** 10.1111/dewb.12157

**Published:** 2017-07-28

**Authors:** Derrick Aarons

**Keywords:** research in emergency, ethics review, research ethics, research protocols, research ethics committee / IRB, ministry of health

## Abstract

Various forms of research are essential in emergency, disaster and disease outbreak situations, but challenges exist including the long length of time it takes to get research proposals approved.

Consequently, it would be very advantageous to have an acceptable model for efficient coordination and communication between and among research ethics committees/IRBs and ministries of health, and templates for expediting (done with speed and efficiency) ethical review of research proposals in emergency and epidemic situations to be used across the Caribbean and in other low and middle income countries.

This project involved a literature search and the interviewing of ministry of health officials, public health practitioners, and research ethics committee/IRB members in Jamaica and St. Lucia, to obtain suggestions for the best model for efficient coordination and communication between research ethics committees (RECs), and developed a template for expediting review of research protocols in epidemic and emergency conditions.

## 1 INTRODUCTION: RESEARCH IN EMERGENCY SITUATIONS

Disasters, emergency situations, and epidemic infections are occurring almost annually, and when they occur, the public expects there will be an emergency response with the quick activation of public service agencies and rapid solutions to the disaster or epidemic infection. However, for the responses to be appropriate, measured, and effective, they need to be informed by the requisite evidence that often can only be obtained by relevant research during the actual emergency or epidemic.^1^

Research, by its very nature, seeks to gain new information for the benefit of society.^2^ In conducting research, the methodologies used will vary widely, but whatever design is used, research that seeks to benefit human beings should give priority to people's health problems and seek to reduce inequities.^3^,^4^ Research in emergency situations will therefore require adequate preparation and anticipatory planning, with the requisite education and training.

In every emergency or epidemic situation, ‘time is of the essence’, and valuable time may be lost particularly when the coordinating body within a country has to undergo preparations in order to function. We saw examples of this during the 2014 – 2016 Ebola virus epidemic in parts of Africa, and the chikungunya virus infection that caused much morbidity in all countries of the Caribbean. We are now witnessing a wave of zika infection with its severe adverse neurological effects in some foetuses and neurotropic effects in some adults.

Research during these situations could be invaluable, and sometimes has highly beneficial outcomes that include preventive vaccines and speedy treatment to minimize harm. As new and emerging epidemic infections are now occurring regularly in the global village, perhaps a permanent committee in each country that is prepared to address matters of research may be one solution to the current challenges for research in epidemic and emergency situations. The aim should be to minimize harm by saving lives as well as the efficient use of all resources.

## 2 RATIONALE FOR THIS PROJECT

Various forms of research are essential in outbreak situations, but procedural challenges such as the long length of time to get research proposals approved present a perennial problem worldwide.^5^ In addition, at the Global Forum on Bioethics in Research that discussed emerging epidemic infections (November 2015), there were repeated calls by various presenters regarding the need for collaboration between various research ethics committees (RECs/IRBs), particularly in epidemic situations. A standardized application form and a template for the ethical review of research proposals in emergency situations therefore needed to be developed, as well as models for collaboration between various research ethics committees in epidemic situations, especially when multi‐centre studies are proposed. This could possibly expedite the conducting of research in public health emergencies, and facilitate the rapid sharing of research outcome data that could be highly beneficial not only to local communities, but also to the global community.

In light of all this therefore, the author utilized the knowledge and expertise existing within the Caribbean to produce templates that can facilitate the rapid conducting of research during epidemic or emergency conditions.

## 3 METHODOLOGY

This project was not conducted as a research project, and so no formal research protocol was written and submitted to a research ethics committee. A literature search was conducted on RECs/IRBs and their procedures in emergency situations in the Caribbean and worldwide, to ascertain whether any prior guidance existed for how RECs should deal with research in emergency and epidemic situations.

As the largest English‐speaking Caribbean country (and situated in the western Caribbean), Jamaica has four (4) functioning research ethics committees and so the Chairs of the 4 RECs were identified as key informants. Other key informants were public health officials in the Jamaican Ministry of Health and a major researcher who also serves on the largest research ethics committee on the island. The Organization of Eastern Caribbean States (OECS) comprise 9 small island countries with St. Lucia being the centre of its governance, and so the latter country was identified as being representative of the smaller islands of the Caribbean for this project. Key informants were identified at St. Lucia's Ministry of Health and from its research ethics committee.

Nine persons were interviewed in Jamaica, and eight persons in St. Lucia. The persons were specifically selected to be interviewed because of their work positions, knowledge of the subject area and experience – to be able to contribute to the knowledge and perspectives being sought. Interviews began with a preamble regarding emerging epidemic infections and the need for research during these epidemics, followed by specific questions.

Eight main questions were asked, and individual responses were subsequently evaluated. The individual responses to each question were tabulated, and those that made the same points in common (coding) were written up. Other individual responses that were not proffered by other respondents were also evaluated for their pertinence in answering the question posed. Those that were pertinent were also included to provide a comprehensive response (collated answer) to each question (see Results).

Further, among the many issues discussed at a retreat workshop of the REC of the Caribbean Public Health Agency (CARPHA) in February 2016 were the components of what would be required for any submission to the committee for research in emergency situations.^6^ That REC is the regional research ethics committee serving its 24 member states in the Caribbean. One outcome of the workshop was their recommendations for the most important subject headings in an application form for research in emergency situations, and guidelines for the content of such research proposals.

All recommendations and data obtained were used to derive the standard application form to research ethics committees/IRBs for research in epidemic or emergency conditions, and a template for communication and collaboration between research ethics committees.

## 4 LITERATURE SEARCH REGARDING RECS AND THEIR PROCEDURES IN EMERGENCY SITUATIONS

Using MEDLINE/Pub Med, Google Scholar and WorldCat databases, literature searches were conducted. Keyword searches for the Caribbean included: ‘Ministries of Health (MOHs) in the Caribbean and epidemic situations’; ‘Ministries of Health in the Caribbean and emergency situations’; ‘Ministries of Health in the Caribbean and policy regarding research in emergency conditions’; ‘Ministries of Health in the Caribbean and policy regarding research during epidemic situations’; ‘Ministries of Health in the Caribbean and procedures for research in emergency conditions’; and ‘Research Ethics Committees in the Caribbean.’ Similar searches were done for worldwide situations, using keywords such as ‘IRBs and emergency situations’; ‘RECs and epidemic situations’; and ‘Research in emergency situations.’

A research of the literature found nothing written specifically on research or research oversight in emergency situations in the Caribbean, or regarding ministries of health in the Caribbean and policy regarding research in emergency conditions. However, publication regarding the HIV epidemic in Latin America and the Caribbean^7^ and the social response were found.^8^ Another article described the work of the ministry of health in Haiti and its cholera surveillance during the Haiti Epidemic.^9^ However, it provided no guidance on how ministries of health in the Caribbean could address policies regarding research and research ethics review during epidemic situations.

Other articles described research endeavours regarding chronic diseases and public health challenges in the Caribbean,^10^ integrity research into policy and programmes,^11^ and HIV/AIDs and violence against women in the Caribbean islands of Barbados and Dominica,^12^ but while they all researched important public health issues in the Caribbean, none contained anything that analytically assessed ministries of health in the Caribbean, or their work in emergency or epidemic or emergency situations, or the role of RECs/IRBs in the process.

A further search was then made regarding the work of research ethics committees in the Caribbean. One article stated that guidelines for RECs exist at national and international levels,^13^ while another stated that having attained a multinational consensus about what the fundamental guidelines should be, RECs are left to interpret the guidelines and devise their own means of implementing them.^14^

Another article addressed research ethics in the Caribbean and capacity building,^15^ while another argued for the establishment of RECs across the Caribbean to ensure that clinical research conformed to the highest scientific and ethical standards.^16^ The upsurge in international biomedical research projects being conducted in Latin America and the Caribbean and the universal validity of ethical principles were stressed by another article, but again no mention was made regarding the role of research ethics committees in ethics review of research, or the particularity of research in epidemic or emergency conditions.^17^

Research did not reveal many publications worldwide with a primary focus on guidance for research ethics review in emergency circumstances, a finding also confirmed by Tansey et al,^18^ however, an article was found related to research in low and middle income countries (LMIC) under humanitarian crisis conditions.^19^ It acknowledged the necessity for research to accurately describe phenomena in humanitarian emergency situations, and to evaluate the effectiveness and appropriateness of interventions. It purported that whilst the 4 basic ethical principles should be upheld in research, their application in emergency situations may differ from non‐emergency situations.

The authors opined that victims of emergency situations are vulnerable populations that need special protection from exploitation, and so technical competency to conduct research in emergency situations should include the ability to conduct a fair risk‐benefit assessment to come up with a risk management plan, while being culturally sensitive to the needs of the victims of a humanitarian crisis. They proposed that in emergency situations the roles of IRBs may have to be modified without compromising the ethical standards that health researchers have globally attempted to achieve.

Another article reviewed specific challenges occurring at the boundary of public health investigations and research, including issues of informed consent and institutional reviews processes,^20^ while another looked at ethical issues arising in the context of research in disasters, but with no reference to ethics review of the research.^21^ Interestingly, one paper proposed a framework for ethics governance that would encourage conversations about ethical issues in the oversight of research rather than the imposition of quasi‐legalistic rules in the context of research being done by Médecins Sans Frontières (MSF).^22^

Related to MSF, another article examined the experience of the Ethics Review Board (ERB) of MSF in addressing the process of review and ethical issues that arose in that context.^23^ It proposed that when multiple ethical reviews of clinical and vaccine trials are to be done in public health emergencies, they should be accompanied by transparent communication between the ethics committees involved, a proposition that was central to the project conducted in the Caribbean.

Another study examined IRB members’ experience in reviewing research protocols using emergency exception from informed consent, and found that those protocols took longer to review than other protocols.^24^ Another reviewing the ethical guidelines related to research in disaster settings concluded that some concepts and terms identified in analyzed guidelines were used in an inconsistent manner and applied in different contexts.^25^ However, it made no recommendations regarding research ethics review.

Guidance for managing ethical issues in infectious disease outbreaks have been issued by the World Health Organization (WHO), and includes guidelines on allocating resources, research during infectious disease outbreaks, and the emergency use of unproven interventions outside of research, but again there was no specific guidance on the ethical review of proposals for research during epidemic and emergency situations.^26^

A training manual by the WHO on ‘Ethics in epidemics, emergencies and disasters: Research, surveillance and patient care’, provides an overview on ethics in epidemics, emergencies, and disasters, and seeks to increase core competencies in research and their ethical implications in emergencies, what qualifies as ‘research’ during emergency responses and would normally qualify for ethics review, and to identify the shortcomings of current normative instruments for use in emergency situations and evaluate alternatives.^27^ However, it does not provide a model for communication and collaboration between research ethics committees during emergency situations, nor a standard application form or template to be used for the ethical review of research during emergency conditions.

The results of our literature review indicate that there is guidance existing regarding emergency situations with regard to operations under surveillance, and some specific guidance for research in emergency situations. However, no publications exist that could be used to guide research committees regarding expediting research in epidemic or emergency conditions, nor on collaborating when multi‐centre research in disaster or emergency situations will be conducted within their jurisdictions.

## 5 RESULTS: THE INTERVIEWS

### 5.1 Interviews in Jamaica:

***Question 1: What are your thoughts on research and research ethics review in emergency conditions?***

**Collated answer (from all respondents in Jamaica) – question 1:**

Bureaucracy, caution, and legal issues provide parameters, and may cause delays in the matter. Rules and guidelines will therefore be necessary to guide the process. A specific template should exist which would be activated during an emergency or epidemic infection. Research ethics committees should be made aware of these special needs (for research in emergency and epidemic infections).

***Question 2: Is collaboration between the various research ethics committees in Jamaica possible?***

**Collated answer (from all respondents) – question 2:**

Whilst collaboration between research ethics committees is possible, hubris is likely to be an issue as each research ethics committee would not wish to regard itself as being subservient to another, or to give up control over its current areas of jurisdiction. There is currently no structure that connects one research ethics committee with another in Jamaica. Whist that at the Ministry of Health in Jamaica should function at the ‘national’ level, not all research proposals submitted to it can be evaluated and processed quickly, and there are significant delays in assessing protocols and so some research applicants actually begin their research before approval is given.

Perhaps an ad‐hoc committee, comprising representatives from all stakeholders that would meet quarterly to plan for its possible response in emergency and epidemic situations.

Decisions made would be communicated back to the respective research ethics committees via their representatives on the ad‐hoc committee. Legal backing would also be needed for this committee.

***Question 3*** **:** ***What, if any, are the impediments?***

**Collated answer – question 3:**

No one committee knows what the other is doing, and so how does one know how they would behave in an emergency situation? Competing universities will have ‘competing’ research ethics committees, which may also function under different institutional factors, rules, and cultural issues. There may also be political considerations. There is no system now for collaboration, and research ethics committees may not be knowledgeable about each other and each other abilities and competencies. Questions such as ‘who decides there is an emergency’ is also important. Consequently, a Terms of Reference would be needed, particularly for emergency situations.

***Question 4. How might these impediments be addressed and removed, if possible?***

**Collated answer – question 4:**

Impediments could be addressed and removed in specially constituted meetings. As each committee wishes recognition for their areas of authority, each would be asked to articulate what they perceive to be impediments to their collaborating on the evaluation of research proposals. Mutual respect between research ethics committees would also have to be fostered for the work that they do. Thus concerns could be met in order to reach consensus. Getting to know each other and how each committee handles research proposals will inspire confidence and respect for each other.

In the medium to long term, Jamaica needs legislation for research with human participants, as what ought to occur can be stipulated in the legislation. Any alternative to legislation would have to be by force or by threat. Researchers should not perceive the process as a ‘stumbling block.’

***Question 5: What do you think would comprise the best ways for these research ethics committees to communicate with each other?***

**Collated answer – question 5:**

Electronic communication, such as a dedicated email group or address for applicant research ethics committees would be desirable. There should also be a master list of contacts and addresses of all research ethics committees, which would be circulated to all of them. A focal point involving a secretariat would also be good, and there should be a formal process involved. Regular reporting should also be a part of the process.

Also, one could commence an institutional framework with a meeting involving representatives (or Chairs) of all the research ethics committees, conceiving a Terms of Reference, and then proceeding from there. Currently there is no interaction between the various research ethics committees in Jamaica. Each representative would then lobby their own committee or institution.

***Question 6: Given your knowledge of this particular Jamaican society, which of the following do you think would be the better model for decision‐making and possible approval of a research project in epidemic infections or emergency situations:***

***A. Individual RECs meet and make independent decisions***

***B. Individual RECs defer evaluations and decision‐making to one committee?***

**Collated answer – question 6:**

Both answer A and B were chosen as the better model for decision‐making for approval of a research project in emergency and epidemic situations. For A, the reasons given included the reality that this is what in fact happens. In this case, RECs should follow a template and simply pay attention to ‘red flags’ as they occur. Institutional politics dictates that none will allow another committee to determine issues of research approval for them. Another rationale is that not all the committees are equal in expertise and training, and so individual committees prefer to make their own decisions based on their own standards.

For choice B, the reasons given were for a special committee that would be constituted for emergency research approval only, and would meet perhaps on a quarterly basis. To achieve more, its composition should be small, e.g. 6–7 persons. These representatives would be ‘high‐powered’ persons who would make decisions on behalf of their own institutions. Another rationale is that timeframe would be greatly reduced for ethical review. The major issue though is that in an epidemic situation, there should be one committee. This committee would be activated under the auspices of the Ministry of Health, and function for emergency situations and epidemic infections only.

There was also a suggestion to have two committees doing the evaluation instead of one, and a secretariat would then inform all the other committees that the research had been approved by two (2) committees.

***Question 7: If your choice is ‘B’, which committee in Jamaica might be best for this – and what are the pros and cons?***

**Collated answer – question 7:**

If the ethical review of research proposals in epidemic and emergency situations is to be done by one committee, that committee would have to be created, as the current ones are ‘tarnished.’ In an emergency, it might not be health‐related alone, and so other ministries, etc., may also have to have representation. There needs to be a ‘national’ committee.

***Question 8: What do you think would be the most efficient and effective way to collaborate research ethics review in epidemic and emergency situations?***

**Collated answer – question 8:**

We need to have an emergency research ethics committee that is activated once an emergency occurs. Appropriate communication with the public will also be crucial, and a specific protocol for such communication would have to be developed. Another possibility is to also conduct a research ethics training workshop to bring all the Chairs of RECs together to form a MOU (Memoranda of Understanding) including how they feel about each other committees making decisions for them to accept, what would be required for them to respect and accept the decisions made by other committees, what type of research decisions they would like to retain for themselves, and the legal ramifications of these decisions for their institutions.

Concurrent reviews of research proposals were also suggested, with corrections being done simultaneously by the pertinent committees. If the epidemic or emergency situation extends to regional involvement, then the ethical review could be conducted through the CARPHA REC. If it is local to Jamaica, then it should really be the Ministry of Health, but their ethics committee currently is not efficient. The most efficient and effective way to collaborate research ethics review in epidemic and emergency situations will require for someone to take the lead, and to arrange for committees to meet and get things going. After the meetings, the discussions could be shared electronically. Patient/community representation should be a part of the discussions.

Another suggestion is for an emergency panel of suitable qualified persons to react to the urgency of the situation, and having representatives of the other committees serving on this emergency committee. The Chairs and their secretariats should be involved. Training of admin secretariats should occur for them to recognize and respond appropriately in epidemic and emergency situations.

### 5.2 Interviews in St. Lucia

***Question 1. In what structured ways, if any, do member countries of the OECS corporate and coordinate in epidemic or emergency situations that affect them?***

**Collated Answer (from respondents in St. Lucia) to Question 1:**

This is done in a number of ways – from planning to the actual response, e.g. by the MOHs – at the Ebola threat, member states came together where individual states did not have the ability of ‘economy of scale’ to address these issues. It also occurs through technical assistance by the OECS Secretariat, PAHO, and CARPHA. There is also COHSOD (CARICOM Ministers of Health) at the higher level, with the possibility of coordination there.

***Question 2. As far as you are aware, have any research projects been conducted in St. Lucia or other countries of the OECS during epidemic or emergency situations?***

**Collated Answer – Question 2:**

Quite a few. The Chik‐V project in St. Lucia, with REC approval, the Dengue fever prevention, the Drug resistance for HIV, figure highly. All came out of a larger epidemic. There is however quite a lot of room for such research, and a lot more can be done in St. Lucia.

The Chik‐V research was conducted in the ‘tail‐end’ of the epidemic. Ethical review and approval was done and given. This is the only one done in relation to an epidemic or emergency situation.

***Question 3. If so, specifically what was the purpose of the research (towards what outcome was the research done), how was it conducted, and were any arrangements made for ethical review beforehand?***

**Collated Answer – Question 3:**

Although not done during an epidemic or emergency situation, there was the Ministry of Health Dengue Fever Prevention Program to evaluate the dengue fever prevention in communities, in order to examine the knowledge, attitudes and practices in two areas of St. Lucia, conducted Sept. – Dec. 2011. This also received ethical review and approval.

***Question 4. If research was done, were there any lessons learnt? If so, what were they?***

**Collated Answer – Question 4:**

Yes, and they would have been well documented in the papers prepared. ‘System issues’ identified. Notably, the absence of a culture of M & E (Monitoring & Evaluation). The requisite ‘base information’ not being recorded, which hinders research. Research must be conducted rapidly to provide information for prevention in a timely manner; research guidelines and protocols must be available and used for training partners – which must be done routinely; and depending on the nature of the disease and scope of the epidemic, a multi‐dimensional research team must be in place, including risk communication.

***Question 5. As far as you are aware, has there been any cooperation or collaborative efforts between the research ethics committee of St. Lucia and other research ethics committees (RECs), particularly within the OECS? RECs within the OECS include the REC for Antigua/Barbuda, the REC for the Commonwealth of Dominica, the REC for Grenada, and the REC for Anguilla.***

**Collated Answer – Question 5:**

Only the collaboration that occurred between the Chairs of Caribbean Research Ethics Committees at a workshop in Barbados in March 2016. Such efforts support stronger collaboration and cooperation, and one can get insights, etc. If multi‐national research, individual review is done without collaboration. The St. Lucia REC has reviewed proposals that are multinational in character, in the past.

***Question 6. If no such collaboration has occurred in the past by your recollection, do you think such collaboration is possible? What would be some challenges or impediments?***

**Collated Answer – Question 6:**

There always are some impediments on the legislative side. The biggest hindrance is getting all islands to the same level with respect to legislation. However, mechanisms exist to address these. We just need to get concurrence. However, implementation becomes the hindrance thereafter, due to issues surrounding legislation.

However, communication lines are open, and we have situations in common. Time‐lines are a challenge; but Skype or meetings are possible. Other impediments include time off from work to attend and cooperate, movement between the islands, as well as money (cost) to move around. Research ethics is in its infancy in some countries and so collaboration is important to the functioning of these RECs. Some of the challenges would be having legislation that is cross‐cutting, and ensuring similarity in the composition of RECs may pose a challenge as each country has its own cadre of professionals. Other possible challenges are that some countries do not have any REC. More than one person (the Chief Medical Officer) reviewing a protocol is desirable.

***Question 7. What do you think would be the best model for collaboration and communication between the RECs in the OECS?***

**Collated Answer – Question 7:**

The best model for collaboration would be annual conferences for the general body of Chairs of RECs, CMOs and Ministers of Health. Other suggestions include conferences, MOUs among the islands – regarding the process to be followed. The regional committee at CARPHA could also suffice. Local committees would only agree to let others evaluate for them if the matters are not sensitive, e.g. GM mosquitoes. Some countries might differ in approach on such matters.

***Question 8. Do you think it is likely that, in epidemic and emergency situations in the OECS, that research ethics committees within the OECS would facilitate one committee reviewing proposed research on their behalf, or would each committee wish to review such research proposals on behalf of their own inhabitants? Please provide reasons for your answer.***

**Collated Answer – Question 8:**

Each committee would want to review on behalf of their own inhabitants, however, in emergency, if a capacity issue arises, then another REC from another jurisdiction could do so. The process however should not be mutually exclusive. It should not be one or the other – both options should be available.

It depends on the scale and nature of the epidemic/emergency. In a situation where immediate actions are required to relieve suffering and death, the review should be done locally as challenges may arise in the timely convening of the regional committee. In less urgent situations and in cases where the scope of the research is regional – a regional committee may suffice, and could also support local ‘buy‐in.’

It may not be ideal to have one committee review on behalf of all countries, as each country has its own cultures, socio‐economic situation, cadre of professionals, and resources. Consequently, the outcomes may vary which might affect each country differently. What the review can do is to facilitate expedited review in the other countries so that they can apply their own unique situation. Every country should review the proposal to take into account local nuances, etc., but can accept a review from another island, in order to expedite the research. It would not need a full committee to review.

### 5.3 Interview Summary

These intensive interviews conducted in Jamaica and St. Lucia provided invaluable suggestions regarding possible communication and collaboration strategies between research ethics committees, and recommended possible models for the oversight of research in epidemic and emergency situations.

## 6 DISCUSSION: THE MODEL FOR REC COLLABORATION AND COMMUNICATION DURING EMERGENCY

Researchers who conduct research with human participants should be fully conversant with the fundamental considerations that underpin research ethics, including the requirement of the ethical review of research by a research ethics committee/IRB prior to the commencement of the research endeavour.^28^ When research is to be done during emergency conditions or epidemic situations however, it is crucial that research proposals be speedily and efficiently assessed so as to effect the rapid conduct of research to hopefully provide relevant solutions.

One example of such a need was the severe acute respiratory syndrome (SARS) outbreak in Toronto in 2003, when clinical researchers complained of delays and missed opportunities for their research protocols due to the need for approval from research ethics boards.^29^ Tansey et al thus proposed a framework to guide departures from normal research ethics reviews during emergencies, utilizing the concepts of proportionate review, enhanced diligence, and expedited review.^30^ However, their proposal did not address the particular issue of how RECs may collaborate in reviewing proposals for research in epidemic or emergency conditions.

RECs customarily conduct concurrent reviews of multi‐centre research proposals, but for research proposed during emergency or epidemic situations, such reviews may hinder rather than aid in speeding up an efficient review of protocols. Further, the role, mandate, and jurisdiction of RECs may vary depending on whether they are based in the public sector (Ministry of Health), academic institutions, or are independent and fully autonomous.

If research is proposed in Jamaica under an emergency situation (for example, post hurricane conditions), then all four RECs might be approached by researchers, and so collaboration would be vitally important. Whilst that at the national level of the Ministry of Health should be paramount, respondents expressed concerns regarding its inefficiencies as well as pointing to the autonomy that academic institutions have over research within their purview. A similar scenario was found in the OECS, where the REC of St. Lucia has the capacity to review research proposals from the nine member states of the OECS. However, if the Caribbean region is hit by an epidemic, then the work by the regional REC provided by CARPHA could be crucial for expediting ethics review of proposed research across the Caribbean. CARPHA has the mandate to respond to the public health priorities of its twenty‐four (24) member states.

This project sought to provide a model for communication and collaboration between research ethics committees to evaluate research during emergency situations when required, and such a model would have been very advantageous to the researchers conducting research in the countries affected by the 2014‐2016 Ebola outbreak, as they were hindered by insufficient dialogue between the RECs and inadequate harmonization of the multiple review processes involved in such urgent situations.^31^ The model of communication and collaboration recommended in this project addressed questions such as ‐ What communication models work best for ministries of health in the Caribbean and research ethics committees? Which would be the most efficient and effective in collaborating on reviews for research in emergency and epidemic situations?

Consequently, the recommended model for collaboration to evaluate any proposed research in epidemic and emergency situations is:

### 6.1 Model

An ‘Ad‐hoc’ research ethics committee ‐ convened specifically for epidemics & emergencies

Composition: 6 – 7 members (Chairs of the main RECs; Ministry of Health; Community)

Communication: Via Chairs of the RECs back to their committees and institutions

Recommendations:

Should have legislative support
Will need secretarial support
Will need Terms of Reference
Initial meeting before any emergency or epidemic
Master list of contact information for all stakeholders
To be implemented under the auspices of the local Ministry of Health
Should function for epidemic and emergency situations only

The model is based on the collation of all the recommendations obtained in all the interviews. This template describes a committee that would be activated in regards to research in epidemic or emergency situations, and would involve a single ‘ad‐hoc’ research ethics committee specifically convened for the purpose. It would be relatively small at 6 ‐7 persons, and comprising representatives from the local ministry of health, the chairs of the pertinent research ethics committees, and any specifically affected or interested parties to be determined locally. Further, community representation and involvement should occur. Research approval decisions or otherwise would be communicated back to the respective research ethics committees via their representatives on this ad‐hoc committee. Legislation and legal provisions/support should also be made for the functioning of this committee.

An initial meeting of the committee should occur in advance of any epidemic or emergency, and a Terms of Reference (TOR) for its function under emergency or epidemic conditions should be prepared.

Electronic communication, which involve a dedicated email group listing and include the address for all the affected research ethics committees, should be made. There should also be a master list of contacts and addresses of all research ethics committees, which would be circulated to all of them.

Secretariat support for the ad‐hoc research ethics committee should occur. An administrative assistant who would receive relevant training should be a short to medium‐term goal.

As the conditions hereby described would apply to possible research in public health epidemic or emergency situations, this committee would be activated under the auspices of the local ministry of health, and function for emergency situations and epidemic infections only.

The local ministry of health would have the responsibility to implement these suggestions and expeditious requirements, particularly when more than one research ethics committee function within its jurisdiction. The recommendations above would meet the WHO statement that flexible approaches are required to harmonize various review processes, and ensure that the various RECs can review the projects simultaneously and share and discuss the review outcomes with each other.^32^

If the epidemic or emergency situation extends across country borders in the Caribbean region, then the ethical review could be conducted expeditiously through the regional research ethics committee provided by CARPHA.

## 7 RECOMMENDATION TO IMPROVE COLLABORATION AND COMMUNICATION BETWEEN RESEARCH ETHICS COMMITTEES IN THE REGION

This could involve an annual workshop for the Chairs of the various RECs in the region, at which the Chairs would discuss, fraternize, get comfortable with and get to know the background expertise of their counterparts and their committees. They would further discuss strategies to foster and support improved communication and collaboration between themselves. The chairpersons would have received the complete listings of all research ethics committees/IRBs existing in the region, so that they may contact any committee or committees they wish.

### 7.1 Templates

Collaborative work that involves developing country based research may be particularly helpful, especially when template documents are available.^33^ The template developed for an application form to be used under the specified conditions addressed questions such as ‐ What are the crucial details for inclusion in applications for research in emergency or epidemic situations? What are the standards to be met? What are research ethics committees looking for? This template is outlined in Appendix 1.

A template checklist for research ethics committees to use in evaluating proposed research in emergency or epidemic situations was also developed. The details of the recommended templates are to be found in Appendix 1.

## 8 CONCLUSION

As no guidelines or publication exist to advise RECs/IRBs regarding efficient and speedy communication and collaboration between themselves regarding proposed research in disaster, epidemic, or emergency conditions, this project has provided a model for so doing. Where more than one REC exists in a country, an ad‐hoc committee specific for addressing research in emergency and epidemic conditions is suggested, with representatives from the pertinent RECs, ministry of health, and affected communities. Legislative authority and other relevant support should be provided for the committee's functioning by the local ministry of health. A master list with contact details of all the RECs in the region should be available to the committee.

An annual workshop involving the chairs of all the REC in the region would aid in improving their communication and collaboration, particularly during regional epidemic conditions. The use of template documents by the RECs was also recommended.

## CONFLICT OF INTEREST

No conflicts declared.

## NOTES

1 Global Forum on Bioethics in Research (GFBR). (2015). *Global Forum on Bioethics in Research (GFBR) meeting on ‘Emerging epidemic infections and experimental medical treatments’*. Annecy, France: GFBR. Nov. 3‐4.

2 Aarons DE. (1995). Research Ethics*. West Indian Med J*; 44(4); 115–8.

3 Aarons DE. (2015). Research: An ethical answer in addressing our people's health problems and inequities*. West Indian Med J*: 64 (Suppl.2); 97–100.

4 Aarons DE. (2016). Family health, public health, and vulnerability in research: A Caribbean perspective*. Redbioetica UNESCO Journal:* July – Dec; 14.

5 Global Forum on Bioethics in Research (GFBR) , *op. cit*. note 1.

6 The Caribbean Public Health Agency. (2016). *Report on 1* ^*st*^ *retreat workshop of the CARPHA REC*. Port of Spain, Trinidad: CARPHA. Feb. 26–28.

7 Caceras CF. (2002). HIV among gay and other men who have sex with men in Latin America and the Caribbean: a hidden epidemic? *AIDS* Dec: Vol. 16; S23–S33.

8 Calleja JM, Walker N, Cuchi P, et al. (2002). Status of the HIV/AIDS epidemic and methods to monitor it in Latin America and the Caribbean region. *AIDS* Dec: Vol 16; S3–S12.

9 Barzilay EJ, Schaad N, Magloire R, et al. (2013). Cholera surveillance during the Haiti Epidemic – The first 2 years. *New England Journal of Medicine*: 368; 599–609.

10 Ferguson TS, Tulloch‐Reid MK, Cunningham‐Myrie CA, et al. (2011). Chronic disease in the Caribbean: strategies to respond to the public health challenge in the region. What can we learn from Jamaica's experience? *WIMJ* July: 60(4); 397–411.

11 Ashley DE, McCaw‐Binns S. (2008). Integrity research into policy and programmes: examples from the Jamaican experience. *WIMJ* Dec: 57(6); 555–61.

12 Allen CF. (2011). Intersections between HIV/AIDS and violence against women: research to develop pilot projects in Barbados and Dominica. *Journal of Eastern Caribbean Studies*: Vol. 36(4); 39–59.

13 Macpherson CC. (1999). Research ethics committees: a regional approach. *Theoretic Medicine and Bioethics* Apr: 20(2); 161–79. 20:161.https://doi.org/10.1023/a:1009989104496

14 Cox‐Macpherson C. (2001). Research Ethics: Beyond the Guidelines*. Developing World Bioethics Journal* May: 1(1); 57–68.

15 Serrano LaVertin D, Linares Parada, AM. (1990). Ethical principles of biomedical research on human subjects: Their applications in Latin America and the Caribbean. *Bulletin of PAHO*: 2494; 469–479.

16 Aarons, *op. cit*. note 2.

17 LaVertin Serrano D, Parada Linares, AM. (1990). Ethical principles of biomedical research on human subjects: Their applications in Latin America and the Caribbean*. Bulletin of PAHO*: 2494; 469–479.

18 Tansey CM, Herridge MS, Heslegrave RJ et al. (2010). A framework for research ethics review during public emergencies. *CMAJ*; 182: 1489–94.

19 Mfutso‐Bengo J, Masiye F, Muula A. (2008). Ethical challenges in conducting research in humanitarian crisis situations*. Malawi Medical Journal* June; 20 (2): 46–9.

20 Calain P, Fiore N, Poncin M et al. (2009). Research ethics and international epidemic response: the case of Ebola and Marburg hemorrhagic fevers*. Public Health Ethics*; 2: 7–29.

21 O'Mathuna. (2010). Conducting research in the aftermath of disasters: ethical considerations*. JEBM*; 3: 65–75.

22 Schopper D, Dawson A, Upshur R, Ahmad A, Jesani A, Ravinetto R, et al. (2015). Innovations in research ethics governance in humanitarian settings. *BMC Medical Ethics*; 16 (1):1–12.

23 Schopper D, Ravinetto R, Schwartz L, et al. Research ethics governance in times of Ebola. (2017). *Public Health Ethics*, 10(1), 49–61. https://doi.org/10.1093/phe/phw039.

24 McClure KB, Delorio NM, Schmidt TA, et al. (2007). A qualitative study of IRB board members’ experience reviewing research proposals using emergency exception from informed consent. *Journal of Medical Ethics* May: 33(5); 289–93.

25 Mezinska S, Kakuk P, Mijaljica G, et al. (2016). Research in disaster settings: a systematic qualitative review of ethical guidelines. *BMC Medical Ethics* Oct. 21: 17(1); 62. BMC series – open, inclusive and trusted201617:62 https://doi.org/10.1186/s12910-016-0148-7.

26 The World Health Organization. (2016). *Guidance for Managing Ethical Issues in Infectious Disease Outbreaks*. Geneva, Switzerland: WHO Press. ISBN 978 92 4 154983 7.

27 WHO. (2015). *Ethics in epidemics, emergencies and disasters: Research, surveillance and patient care*. Geneva, Switzerland: World Health Organization. ISBN 978 92 4 154934 9.

28 Aarons DE. (2003). Issues in Bioethics: Teaching Research Ethics. *West Indian Medical Journal*: 52(2); 145–50.

29 Tansey et al, *op. cit*. note 1, p. 1489 .

30 Ibid: 1489.

31 Schopper et al (2017), op. cit. note 2, p.51–52.

32 World Health Organization. (2014). WHO Consultation on Potential Ebola Therapies and Vaccines: Background Document for Participants. Geneva, Switzerland: WHO Press.

33 Lang TA, White N.J, Hien TT, et al. (2010). Clinical research in resource‐limited settings: Enhancing research capacity and working together to make trials less complicated. *PLoS Negl Trop Dis* 4(6): e619. doi.org/https://doi.org/10.1371/journal.pntd.0000619.

## APPENDIX 1

### 1.1

***TEMPLATE***:

**RESEARCH IN EPIDEMIC EMERGENCY – APPLICATION FORM**

TITLE OF THE PROPOSED RESEARCH: ……………………………………………………………………..

NAME and CONTACT DETAILS (Address, Telephone and E‐mail) of ALL the INVESTIGATORS, COLLABORATORS, AND/OR *SUPERVISORS* (starting with the principal investigator):

(Indicate which parts of the protocols each investigator will be responsible for, who will actually carry out if any procedure on participants, and where appropriate, what training they have had):

……………………………………………………………………………………………………………………

**SITE/S OF RESEARCH** (Attention should be paid to the facilities available for participants’ comfort, and availability of emergency procedures in the event of an unanticipated occurrence): …………………………….………………………………………………

ESTIMATED NUMBERS OF RESEARCH PARTICIPANTS TO BE ENROLLED:……………

PROPOSED DURATION OF THE STUDY: ……………………….…………………………………..

**A SUMMARY OF THE PROPOSED STUDY** — not more than 250 words and should include:

- The hypothesis and scientific basis or justification for the study
- The usefulness and significance of the study
- An outline of the study design
- An indication of steps taken to ensure and maintain confidentiality
- An assessment of the benefits to participants (and/or groups in the community or the entire community) and the risks

………………………..………………….(Signature of the Principal Investigator) ………………………………………..……….(Date)

***TEMPLATE:***

**RESEARCH IN EPIDEMIC EMERGENCY:**

**CHECKLIST FOR DOCUMENTS REQUIRED FOR PROTOCOL SUBMISSION**

1. COMPLETED APPLICATION FORM
2. **THE ABSTRACT** (Summary of the Proposed Study – not more than 300 words)
3. THE PROJECT PROPOSAL/PROTOCOL
4. THE INFORMED CONSENT FORM

**Note** **:** **A.** **THE PROJECT PROPOSAL** must include:

- An Introduction and Background information on the research topic
- A clear statement of the Objectives of the research proposal
- The Justification for the research (This should include a review of the current knowledge from the literature on the topic, with an explanation why this project is necessary, and how it will contribute to the overall knowledge in this area)
- Materials and methods. These include:
  ‐ Details of procedures to be performed (e. g. volume of blood, the frequency, timing, and possible site of the blood‐taking; any drug administration, physiological measures, etc.)
  ‐ Which procedures may cause pain and/or discomfort for research participants, which are new (i.e. experimental), and which are routine procedures that would have been done on the participants even if they were not involved in the study.
  ‐ Choice of participants, inclusion and exclusion criteria, number of participants (and a justification for that number), any controls, etc.
  ‐ A statement that participants were selected only because of the specific problem under investigation, and not because of their easy availability, diminished autonomy, or any social bias.
- The name, address, telephone and email address of a contact person
- Procedures for obtaining informed consent, including statements that the researcher/s will read the informed consent form to the participant or his/her legal guardian and will provide that person with a copy of the form, that questions from the person will be invited, and that all efforts will be made to ensure that s/he understands its content before the seeking of consent
- A copy of the informed consent form and recruitment posters (see details below)
- Methods to protect the confidentiality of participants, and methods to ensure that a participant who opts out of the research is well protected as far as health care delivery is concerned and not disadvantaged in any way.
- Details of Methods for data collection, analysis and secure storage
- Assumptions made
- Relevant references (i.e. literature citation)
- A copy of any questionnaires to be administered

B. **THE ABSTRACT/SUMMARY OF THE PROPOSED STUDY** – should include:

Summary of the research question
The hypothesis and scientific basis or justification for the Study
The usefulness and significance of the Study (the potential value of the research for public health, etc.)
The population and interventions involved
An Assessment of the benefits (to participants and/or groups in the community or to the entire community), and the risks for participants
An Outline of the Study design, methods, and main outcomes
Names of participating institutions and countries
An indication of steps taken to ensure and maintain confidentiality.

